# Head-to-head comparison of Sonazoid and SonoVue in the diagnosis of hepatocellular carcinoma for patients at high risk

**DOI:** 10.3389/fonc.2023.1140277

**Published:** 2023-03-15

**Authors:** Jiayan Huang, Ling Gao, Jiawu Li, Rui Yang, Zhenpeng Jiang, Min Liao, Yan Luo, Qiang Lu

**Affiliations:** ^1^ Department of Ultrasound, West China Hospital of Sichuan University, Chengdu, China; ^2^ Department of Ultrasound, Chengdu BOE Hospital, Chengdu, China

**Keywords:** contrast agent BR1, Sonazoid, contrast media, liver neoplasms, diagnosis

## Abstract

**Objectives:**

To compare the diagnostic efficacy of SonoVue-enhanced and Sonazoid-enhanced ultrasound (US) for hepatocellular carcinoma (HCC) in patients at high risk.

**Methods:**

Between August 2021 and February 2022, participants at high risk for HCC with focal liver lesions were enrolled and underwent both SonoVue- and Sonazoid-enhanced US. Vascular-phase and Kupffer phase (KP) imaging features of contrast-enhanced US (CEUS) were analyzed. The diagnostic performance of both contrast agent-enhanced US according to the CEUS liver imaging reporting and data system (LI-RADS) and the modified criteria (using KP defect instead of late and mild washout) were compared. Histopathology and contrast-enhanced MRI/CT were used as reference standards.

**Results:**

In total, 62 nodules, namely, 55 HCCs, 3 non-HCC malignancies and 4 hemangiomas, from 59 participants were included. SonoVue-enhanced US had comparable sensitivity to Sonazoid-enhanced US for diagnosing HCC [80% (95% confidential interval (CI): 67%, 89.6%) versus 74.6% (95% CI: 61%, 85.3%), *p* = 0.25]. Both SonoVue and Sonazoid-enhanced US achieved a specificity of 100%. Compared with CEUS LI-RADS, the modified criteria with Sonazoid did not improve sensitivity for HCC diagnosis [74.6% (95% CI: 61%, 85.3%) versus 76.4% (95% CI: 63%, 86.8%), *p* = 0.99].

**Conclusions:**

Sonazoid-enhanced US had comparable diagnostic performance to SonoVue-enhanced US for patients with HCC risk. KP did not considerably improve the diagnostic efficacy, whereas KP defects in atypical hemangioma may be pitfalls in diagnosing HCC. Further studies with larger sample sizes are needed to further validate the conclusions in the present study.

## Introduction

Hepatocellular carcinoma (HCC) is the fifth most commonly diagnosed cancer worldwide and ranks second in terms of cancer-related deaths (1, 2). Contrast-enhanced imaging modalities play a pivotal role in diagnosing HCC. In addition to contrast-enhanced MRI and CT, contrast-enhanced ultrasound (CEUS) is recommended for the characterization of focal liver lesions (FLLs) by established guidelines (3–6). Moreover, CEUS has been demonstrated to be an effective tool in diagnosing HCC (7–9). In clinical scenarios, contrast agents widely used in characterizing FLLs include pure blood-pool agents (PBA), e.g., SonoVue, and combined blood-pool/Kupffer cell agents (KPA), e.g., Sonazoid. Imaging manifestations differ with the use of PBA and KPA.

The CEUS Liver Imaging Reporting and Data System (LI-RADS) released by the American College of Radiology was developed to improve the diagnostic accuracy for HCC and to facilitate communication among radiologists and between radiologists and other physicians (10). Since the criteria were launched, CEUS LI-RADS has been demonstrated to be an efficient tool in the characterization of hepatic lesions in patients at risk for HCC, particularly given its high specificity for HCC of the LR-5 category (8, 11, 12). However, the current version of CEUS LI-RADS is recommended only for application in PBA but not in KPA. Compared with that on PBA-enhanced US images, liver parenchymal enhancement on KPA-enhanced US images can persist for at least 2 hours *via* the phagocytosis of microbubbles by Kupffer cells (13, 14). Whether PBA- and KPA-enhanced US have the same diagnostic power remains unclear. It was reported that PBA-enhanced US gradually showed unsatisfactory enhancement in the late phase due to the degradation of contrast agents, while the liver parenchyma showed stable enhancement on KPA-enhanced US (15). Phagocytosis of KPA by Kupffer cells, which are present in large numbers in the liver (16), may provide additional enhancement by Kupffer cell uptake and have potential impacts on imaging appearance (17). Moreover, several studies found that HCCs without definite washout through all vascular phases may show hypoenhancement in the Kupffer phase (KP) (15, 18, 19). In that respect, KP is expected to improve the sensitivity of CEUS for HCC in high-risk patients due to decreased or absent Kupffer cells in malignancies (18, 20). Meanwhile, KPA-enhanced US also has its limitations, such as pseudoenhancement of KPA-enhanced US for hyperechoic nodules with a higher mechanical index, in some circumstances (18, 21).

Until now, the imaging manifestations of PBA, as well as their diagnostic performances for HCC, have not been fully evaluated or compared to those of KPA-enhanced US. Moreover, evidence that the current version of CEUS LI-RADS can also be extended to KPA-enhanced US remains insufficient. Herein, we conduct a head-to-head comparative study to further evaluate the imaging characteristics of PBA- versus KPA-enhanced US, as well as their diagnostic performance for HCC. Additionally, the possibility of extending CEUS LI-RADS for KPA is investigated.

## Material and methods

This study was approved by the institutional ethics committee and is registered in the Chinese Clinical Trial Registry (Clinical trial number: ChiCTR2000039018). Written informed consent was obtained from each participant.

### Patient selection

Between June 2021 and January 2022, patients with focal hepatic observations by screening or diagnostic US, CT or MRI were consecutively recruited in a tertiary academic medical center. The inclusion criteria were as follows: (1) patients aged ≥18 years; (2) patients with cirrhosis of any cause and/or chronic hepatitis B; and (3) patients who agreed to undergo both SonoVue and Sonazoid CEUS examination and signed an informed consent form. The exclusion criteria were as follows: (1) more than 3 FLLs or diffuse hepatic observations; (2) images with poor quality due to the conditions of patients; and (3) without pathological results either from surgery or biopsy or contrast-enhanced CT/MRI images.

### US examination

Conventional grayscale and contrast-enhanced US (CEUS) examinations were performed by using a Philips EPIQ7 (Philips Healthcare, Bothell, WA, USA) equipped with a C5-1 MHz curved probe or a Mindray Resona 7 (Mindray Medical Solutions, Shenzhen, China) mounted with an SC5-1 U curved probe. All lesions were clearly displayed on B-mode US, and the boundary, echogenicity and sizes of the masses were recorded. All CEUS examinations were performed with a dual screen format. Bolus injection of ultrasound contrast agent (UCA) was administered *via* the antecubital vein according to the manufacturer’s recommendations with 1.2-2.4 mL of SonoVue (Bracco, Milan, Italy) and 0.6-0.8 mL of Sonazoid (GE Healthcare, Milwaukee, WI, USA). The timer was started as the injection of the UCA was completed. Mechanical indices of less than 0.1 and 0.18-0.21 were used for SonoVue and Sonazoid CEUS examinations, respectively. The target lesion and surrounding liver parenchyma were imaged continuously during the initial 60 seconds and intermittently recorded for 5 minutes or longer (22). Ten minutes after injection of Sonazoid, KP images were imaged for several seconds. SonoVue-enhanced US and Sonazoid-enhanced US were carried out on the same day, and Sonazoid-enhanced US was performed at least half an hour after SonoVue-enhanced US. A more detailed CEUS examination protocol is presented in **Supplementary material 1**, and the settings of CEUS with the use of UCAs are summarized in **Supplementary Table 1**.

### Contrast-enhanced US imaging analysis

Imaging data were organized as separate files with information deidentified by the operator (L.G., the radiologist who performed the US examination with five years of experience in liver CEUS). Another two reviewers (Q.L. and J.W.L., with 15 years and 7 years of experience in liver CEUS, respectively) who were blinded to the diagnosis according to reference standards and laboratory results independently reviewed the CEUS examinations. The SonoVue and Sonazoid CEUS images were packed as two separate documents. The readers reviewed SonoVue or Sonazoid image documents randomly with a 10-day interval between each other to avoid any effects of image interference by either agent. Specifically, for both contrast agents, AP enhancement and pattern, presence, timing, and degree of washout were documented. The KP enhancement of Sonazoid was also documented. The category of each nodule according to CEUS LI-RADS version 2017 was applied for both SonoVue and Sonazoid images. Final conclusions of the aforementioned imaging features and lesion categories were obtained by negotiation between the two reviewers. If no consensus was reached, arbitration from a blinded expert radiologist (Y.L., with 18 years of liver CEUS experience) was performed.

### Statistical analysis

All analyses were based on individual liver nodules rather than each patient. The CEUS characteristics of the two UCAs in the vascular phases were compared by using the Pearson’sχ^2^ test or Fisher’s exact test where appropriate. Estimated values of sensitivity, specificity, and accuracy of contrast agents by using CEUS LI-RADS version 2017 criteria or the modified criteria (using KP defect as an alternative to late and mild washout in CEUS LI-RADS) in diagnosing HCC were compared by using the McNemar test. The weighted κ value was used to assess the interobserver agreement of imaging characteristics and CEUS LI-RADS classifications of the nodules. A *p* value less than 0.05 indicated a significant difference. Statistical analyses were performed by using MedCalc 20.027 (MedCalc Software, Ostend, Belgium).

## Results

### Patients and liver nodule characteristics

**Figure 1:**
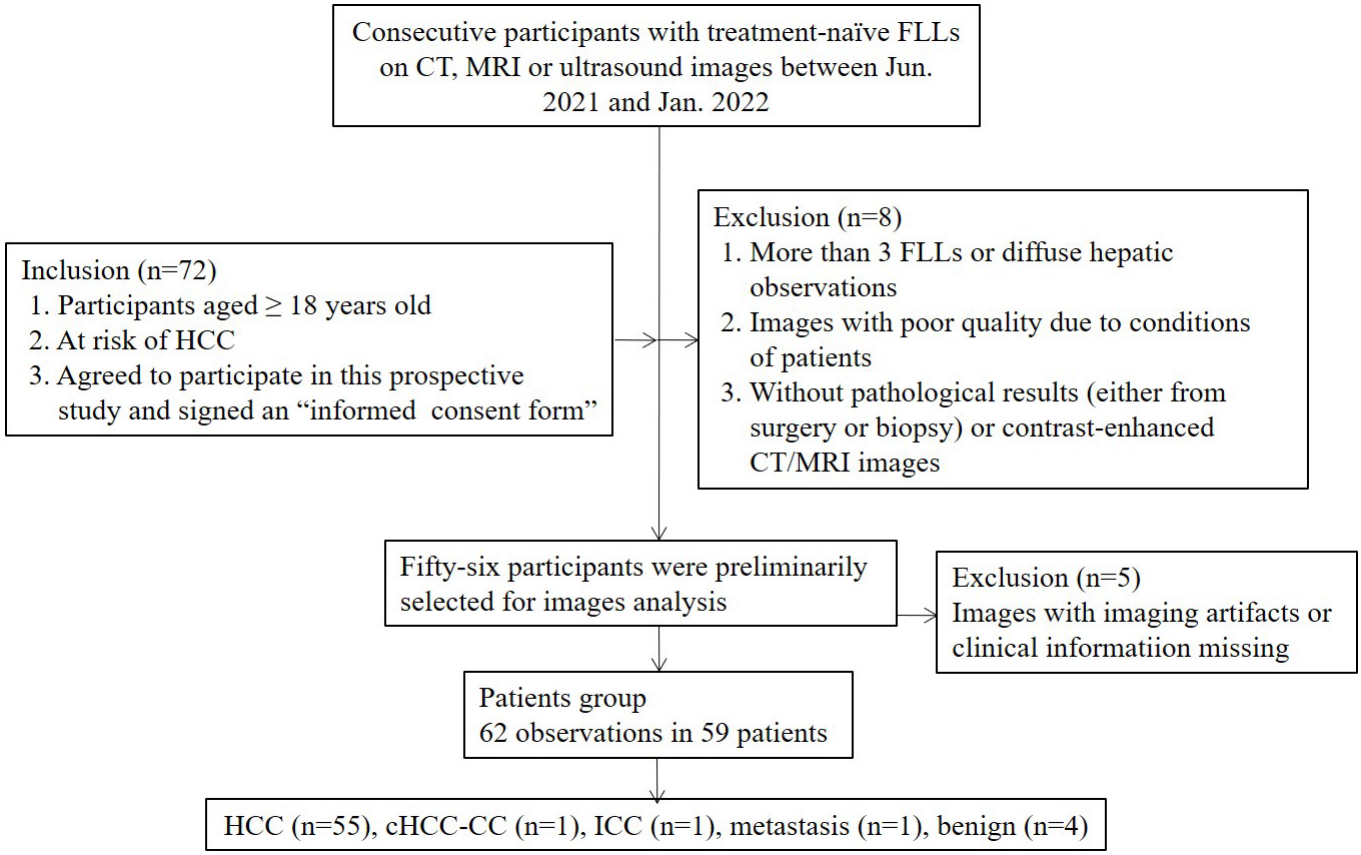
Study flow diagram. FLLs, focal liver lesions; HCC, hepatocellular carcinoma; cHCC-CC, combined hepatocellular-cholangiocarcinoma; ICC, intrahepatic cholangiocarcinoma; CT, computerized tomography; MRI, magnetic resonance imaging.

On the basis of the selection criteria, a total of 62 nodules in 59 patients were included (**Figure 1**). Three patients had two nodules each. The clinical features of the participants and target lesions are listed in **Table 1**. Of the 59 patients (mean age, 54 years ± 11.8), 41 (83.1%) were men. The mean size of observations was 3.5 cm ± 2.3. Histopathologic tissue analyses were obtained in 58 of 62 nodules (93.5%), including 53 HCCs, one hemangioma, one high-grade dysplastic nodule (HGDN), one combined hepatocellular cholangiocarcinoma (cICC-HCC), one intrahepatic cholangiocarcinoma (ICC) and one metastasis. Two hemangiomas and two HCCs were diagnosed by contrast-enhanced MRI/CT. Hepatitis B virus (HBV) infection (86.4% [51/59]) was the major cause of chronic liver disease, and 61.1% (36/59) of patients were simultaneously afflicted with HBV and cirrhosis. Of the 53 HCCs confirmed by histopathology, 64.2% (34/53) were composed of moderately differentiated (MD) HCCs, followed by 30.2% (16/53) of poorly differentiated (PD) and 3.8% (2/53) of well-differentiated (WD) HCCs.

**Table 1:** Clinical and pathologic Information.

Characteristic	Result
Mean age (y)*	54 ± 11.8 (51-57)
Sex	
Men	49 (83.1)
Women	10 (16.9)
Mean nodule size (cm)*	3.5 ± 2.3 (1.0-10.5)
Liver disease etiology	
HBV	51 (86.4)
Cirrhosis	40 (67.8)
HBV + cirrhosis	36 (61.1)
History of HCC	5 (8.5)
Fibrosis stage	
S1	4 (7.4)
S2	4 (7.4)
S3	13 (24.1)
S4	33 (61.1)
NA	5 (9.3)
Pathologic Analysis	
HCC	53 (85.5)
Well-differentiated	2
Moderately-differentiated	34
Poorly-differentiated NA	16 1
Hemangioma	1 (1.6)
DN	1 (1.6)
cHCC-CC ICC	1 (1.6) 1 (1.6)
Metastasis	1 (1.6)
Contrast enhanced CT or MRI	
Hemangioma	2 (3.2)
HCC	2 (3.2)

Unless otherwise indicated, data are liver nodules (n = 56) or patients (n =51) and data in parentheses are percentages. Mean data are ± standard deviation. HCC, hepatocellular carcinoma; DN, dysplastic nodule; cHCC-CC, combined hepatocellular-cholangiocarcinoma; ICC, intrahepatic cholangiocarcinoma; HBV, hepatitis B virus; NA, not available; CT, computed tomography; MRI, magnetic resonance imaging.

*Data in parentheses are range.

### Reference standard

Pathological results and contrast-enhanced CT/MRI diagnosis were used as reference standards. Among all observations, 93.5% (58 of 62) were diagnosed by histopathology *via* surgery (n=57) or biopsy (n=1). Two hemangiomas and 2 HCCs were diagnosed as LR-1 and LR-5, respectively, by contrast-enhanced CT or MRI according to CT/MRI LI-RADS version 2018 (23, 24). The liver backgrounds of the participants were evaluated by pathologic analysis and staged by Scheuer fibrosis staging in patients undergoing surgery.

### Imaging characteristics of contrast-enhanced US

The main CEUS characteristics based on both contrast agents are shown in **Tables 2**, **3**.

**Table 2:** Comparison of characteristics on arterial phase at Sonovue and Sonazoid-enhanced ultrasound.

Variable	Nonrim APHE		Rim APHE		Peripheral Globular Enhancement		No APHE	
	Sonovue	Sonazoid	Sonovue	Sonazoid	Sonovue	Sonazoid	Sonovue	Sonazoid
HCC (n=55)	54 (98.2)	54 ((98.2)	1 (1.8)	1 (1.8)	0 (0)	0 (0)	0 (0)	0 (0)
Non-HCC malignancy (n=3)	2 (66.7)	1 (33.3)	1 (33.3)	2 (66.7)	0 (0)	0 (0)	0 (0)	0 (0)
Benign lesions (n=4)	0 (0)	1(25)	1 (25)	1(25)	2 (50)	2(50)	1 (25)	0 (0)

Data are numbers of nodules with percentage in parentheses. APHE, arterial phase hyperenhancement; HCC, hepatocellular carcinoma.

**Table 3:** Comparison of Washout and Kupffer phase features according to contrast agent used.

Variable	Late (≥60 s) and Mild Washout		Early Washout (< 60 s)		No Washout		Marked Washout (≤120 s)		Kuppfer Phase Filling defect
	Sonovue	Sonazoid	Sonovue	Sonazoid	Sonovue	Sonazoid	Sonovue	Sonazoid	
HCC (n=55)	44 (80)	41 (74.5)	11 (20)	12 (21.8)	0 (0)	2 (3.6)	1 (1.8)	0 (0)	54 (98.2)
Non-HCC malignancy (n=3)	0 (0)	0 (0)	3 (100)	3 (100)	0 (0)	0 (0)	3(100)	3(100)	3 (100)
Benign lesions (n=4)	0 (0)	0 (0)	0 (0)	0 (0)	4 (100)	4 (100)	0 (0)	0 (0)	3 (75)

Data are numbers of nodules with percentage in parentheses. HCC, hepatocellular carcinoma.

#### Arterial phase

**Figure 2:**
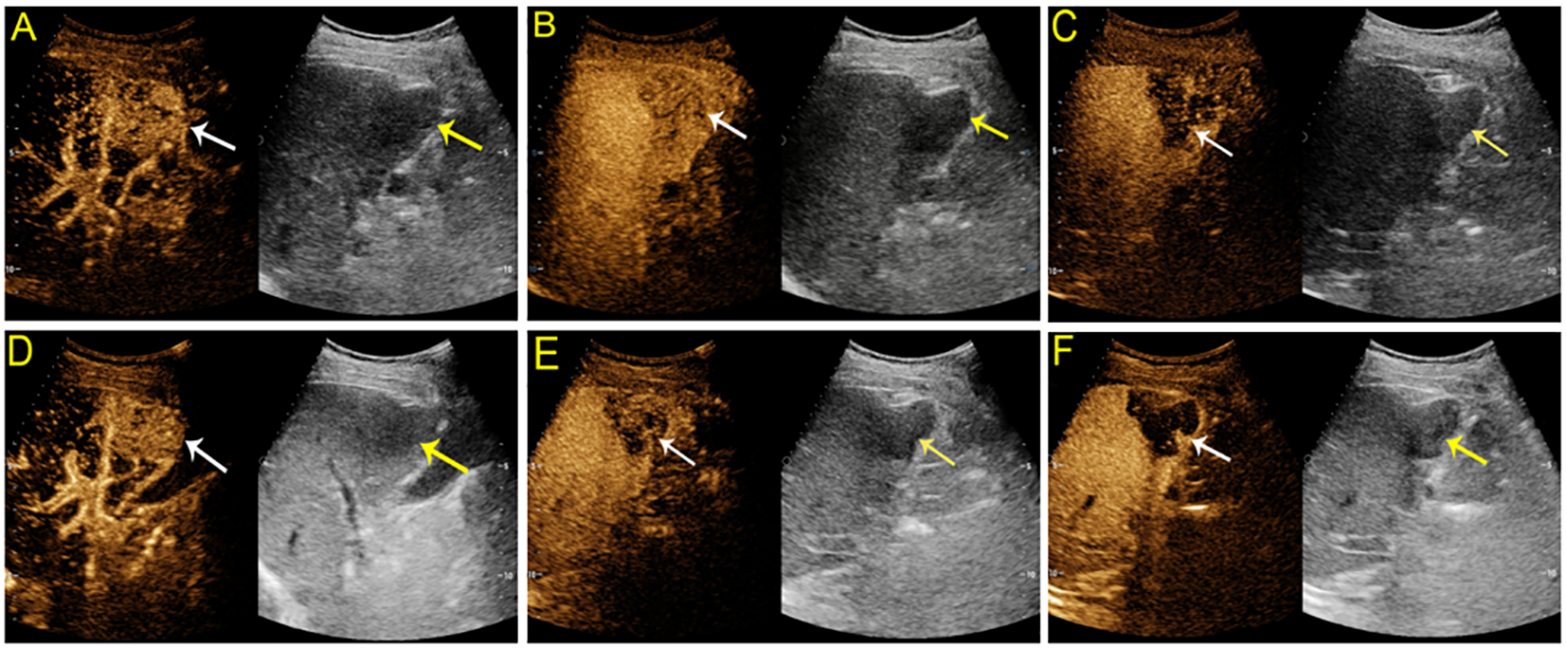
Contrast-enhanced US images from a 68-year-old man with chronic hepatitis B. A hypoechoic mass measuring 5 cm was detected in segment V of the liver. On SonoVue enhanced-US, the mass showed nonrim arterial phase hyperenhancement (APHE) **(A**, white arrow) followed by early washout in thirty-six seconds **(B**, white arrow) and hypoenhancement in the late phase **(C**, white arrow). On Sonazoid-enhanced US, the mass manifested nonrim APHE **(D)**, white arrow) and early washout **(E**, white arrow) followed by an enhancement defect in the Kupffer phase **(F**, white arrow). The mass confirmed intrahepatic cholangiocarcinoma by histopathology.

**Figure 3:**
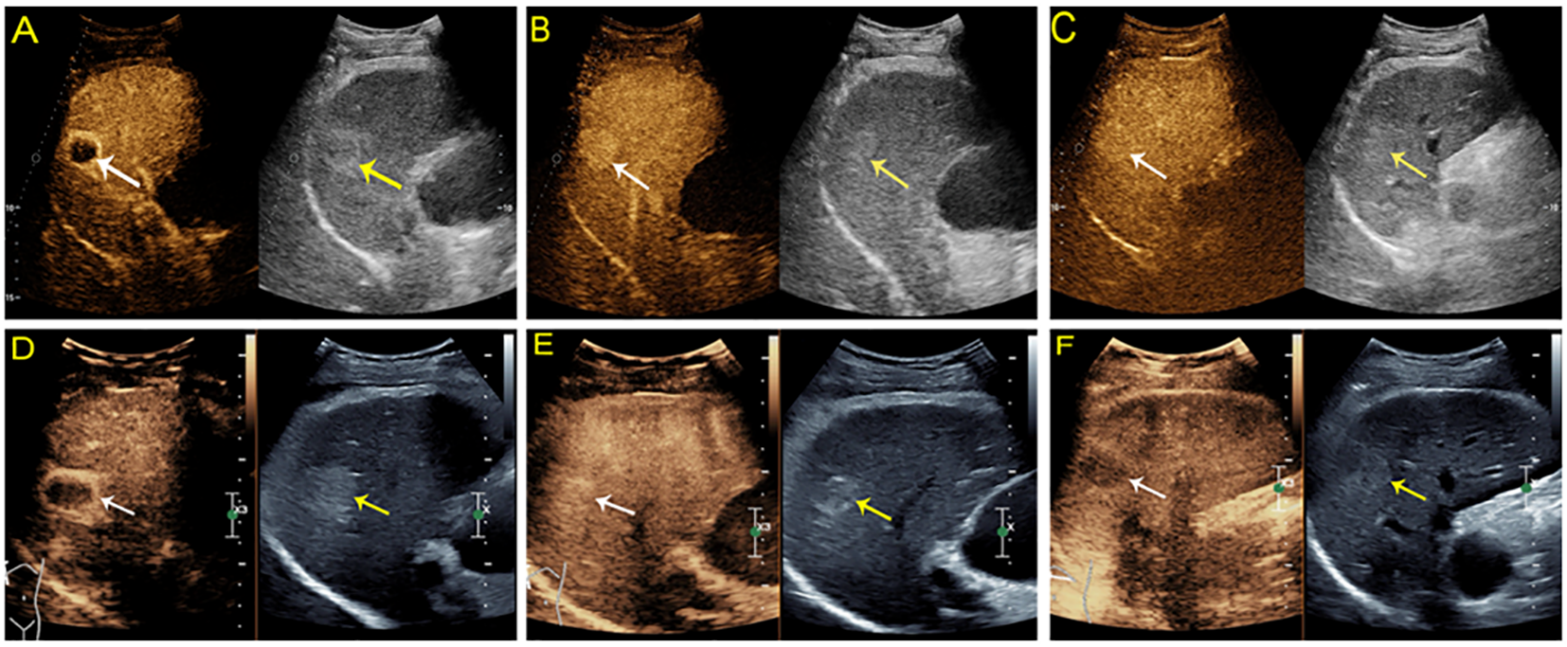
Contrast-enhanced US images from a 51-year-old man with chronic hepatitis B. A hyperechoic solid lesion measuring 2.9 cm in segment VI of the liver was detected on baseline US. On SonoVue enhanced-US, the nodule showed rim arterial phase hyperenhancement (APHE) (**A**, white arrow) followed by homogeneous hyperenhancement in two minutes and thirty seconds (**B**, white arrow) and iso- to mild hyperenhancement in the late phase (**C**, white arrow). On Sonazoid-enhanced US, the nodule appeared to have rim APHE (**D**, white arrow) and iso- to mild hyperenhancement in two minutes (**E**, white arrow), followed by hypoenhancement in ten minutes and nineteen seconds in the Kupffer phase (**F**, white arrow). The nodule was confirmed to be a hemangioma by contrast-enhanced MRI seven months later.

**Figure 4:**
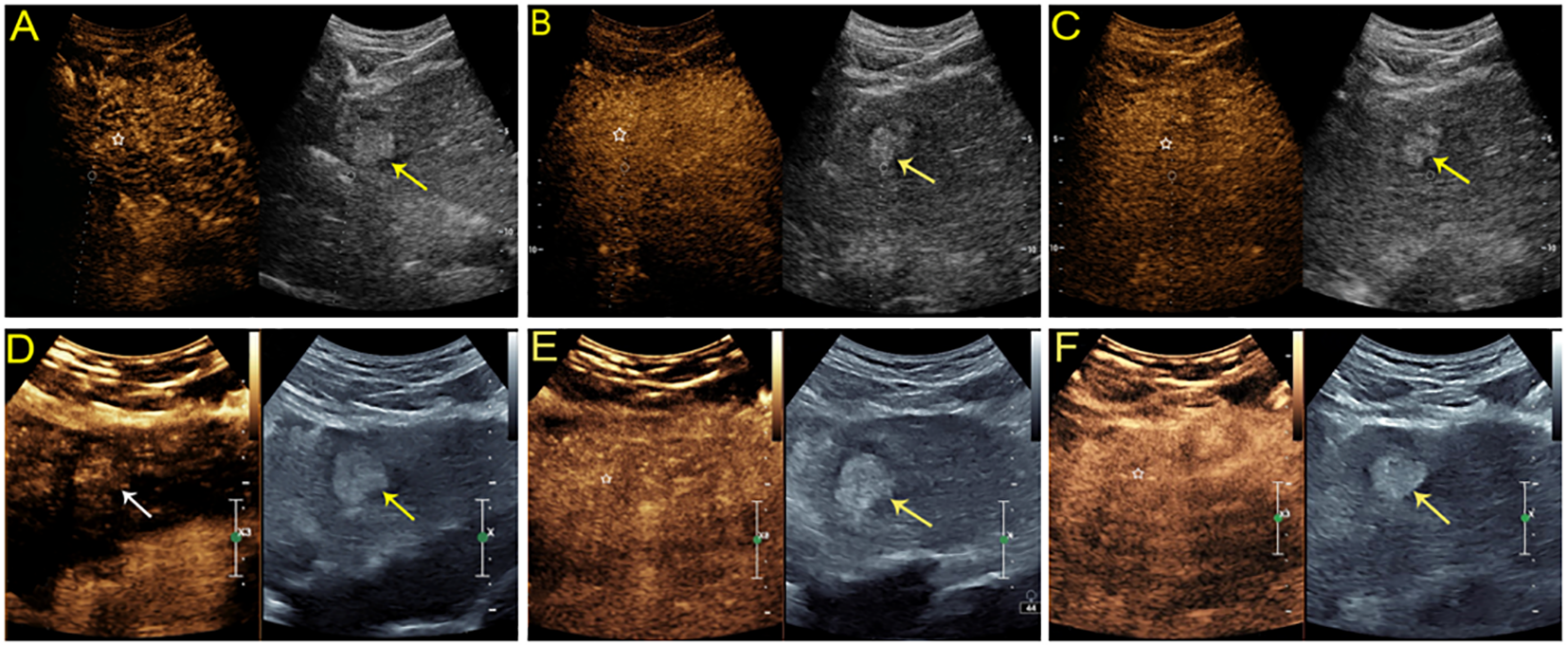
Sonographic images from a 54-year-old man with chronic hepatitis B. A hyperechoic mass measuring 2.8 cm was found in the left lobe of the liver. On SonoVue-enhanced US, the mass showed isoenhancement through all vascular phases without definite washout (**A-C**, white star). On Sonazoid-enhanced US, the mass was slightly hyperenhanced in the arterial phase (**D**, white arrow) without obvious washout in either the subsequent vascular phase (**E**, white star) or defect in the Kupffer phase (**F**, white star). The lesion was confirmed to be a high-grade dysplastic nodule by histopathology.

There was no difference in the arterial phase hyperenhancement (APHE) pattern between SonoVue and Sonazoid CEUS. Fifty-four (98.2%) HCCs manifested nonrim APHE, and one (1.8%) had a rim-like APHE. For the three non-HCC malignancies, the metastasis displayed rim APHE, and ICC (**Figure 2**) showed nonrim APHE on both UCA imaging. However, cICC-HCC demonstrated inhomogeneous APHE on SonoVue-enhanced US but rim-like APHE on Sonazoid-enhanced US. Among the benign observations, 2 hemangiomas manifested peripheral nodular and centripetal enhancement; another hemangioma showed rim APHE (**Figure 3**). In the case of HGDN, the lesion presented isoenhancement through all vascular phases on SonoVue-enhanced US, whereas on Sonazoid-enhanced US, it showed hyperenhancement in the AP without obvious washout through the subsequent vascular phases or defect in the KP (**Figure 4**). The interobserver agreement of the AP enhancement pattern of liver observations was good for both SonoVue (κ= 0.75, 95% confidence interval [CI]: 0.45, 1.0) and Sonazoid (κ= 0.71, 95% CI: 0.35, 1.0) images (**Supplementary Table 2**).

#### Washout

The washout features of hepatic observations are presented in **Table 3**. Washout was observed in all malignancies (100%, 58/58) on SonoVue-enhanced US but 96.6% (56 of 58) on Sonazoid-enhanced US. Two HCCs did not show washout on Sonazoid images. Late (≥60 seconds) and mild washout were observed in 80% (44/55) and 74.5% (41/55) of HCCs on SonoVue-enhanced US and Sonazoid-enhanced US, respectively. In addition, none of the non-HCC malignancies or benign nodules showed late or mild washout in the vascular phase. Early washout was detected in 20% (11/55) and 21.8% (12/55) of HCCs on SonoVue-enhanced US and Sonazoid-enhanced US, respectively. All non-HCC malignancies showed early washout and marked washout with both UCAs. One HCC presented marked washout within 120 seconds on SonoVue-enhanced US but not on Sonazoid-enhanced US. In addition, no benign lesion manifested washout on either SonoVue or Sonazoid images in the vascular phase. No significant difference was detected in the washout pattern between SonoVue-enhanced US and Sonazoid-enhanced US. The interobserver agreements of the washout time and degree were good for both UCA imaging methods, with κ values of 0.82 (95% CI: 0.64, 0.99) for SonoVue and 0.78 (95% CI: 0.62, 0.94) for Sonazoid (**Supplementary Table 2**).

#### Kupffer phase

KP defects were observed in 98.2% (54/55) of HCCs and all non-HCC malignancies. Of those two HCCs without washout through the vascular phase, one manifested hypoenhancement in the KP, whereas another remained isoenhanced. All hemangiomas showed hypoenhancement in the KP, but the dysplastic nodule continuously showed enhancement. The estimate of KP defects of Sonazoid was almost perfect, with a κ value of 0.91 (95% CI: 0.86, 1.0) between reviewers (**Supplementary Table 2**).

### Imaging characteristics of HCC according to pathological differentiation and tumor size

**Figure 5:**
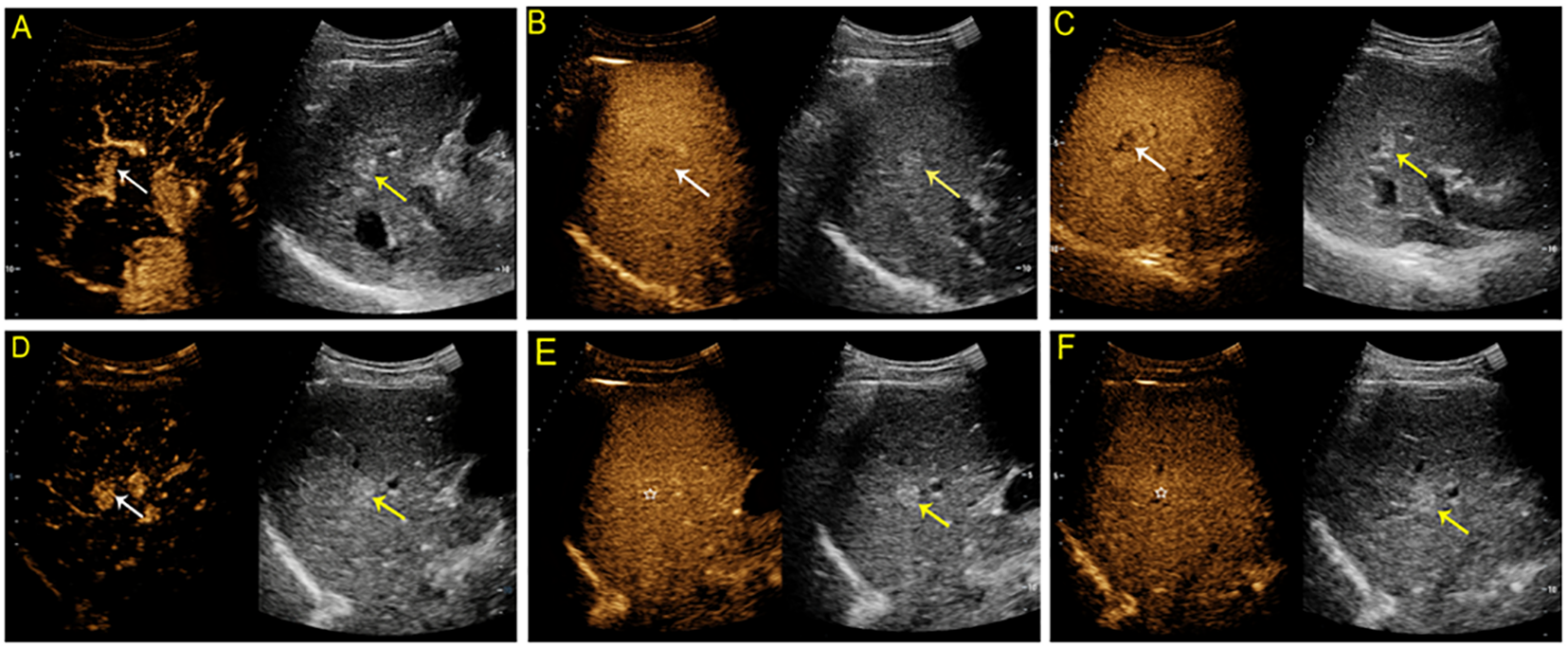
Contrast-enhanced US study of a 54-year-old man with a hyperechoic solid lesion measuring 1.3 cm in segment V of the liver. On SonoVue-enhanced US, the lesion showed arterial hyperenhancement (APHE) (**A**, white arrow) followed by mild washout in one minute and thirty-nine seconds (**B**, white arrow) and in the late phase (**C**, white arrow). On Sonazoid-enhanced US, the lesion showed APHE (**D**, white arrow) followed by isoenhancement in both the vascular phase (**E**, white star) and Kupffer phase (**F**, white star). The lesion was confirmed to be a well-differentiated hepatocellular carcinoma by tissue postoperative histopathological analysis.

The comparison of CEUS characteristics of HCCs between SonoVue and Sonazoid-enhanced US according to pathological differentiation and tumor size are summarized in **Table 4**. One WD HCC (1/2, 50%) showed nonrim APHE followed by late and mild washout on both SonoVue and Sonazoid imaging. However, the other WD HCC illustrated nonrim APHE without wash-out in the vascular phase or KP on Sonazoid-enhanced US (**Figure 5**). At both SonoVue and Sonazoid-enhanced US, most of the MD HCCs (94.1% [32/34]) manifested nonrim APHE, while 2 MD HCCs (5.9% [2/34]) displayed rim APHE. There was no significant difference between SonoVue and Sonazoid imaging referring to the onset of washout in MD HCCs. Of note, one MD HCC (1/34, 2.9%) showed hypoenhancement in the portal and late phases on SonoVue enhanced US but isoenhancement in the corresponding phases and KP on Sonazoid-enhanced US. In addition, all PD HCCs showed the same CEUS manifestations with the use of both contrast agents, and all of them presented hypoenhancement in the KP.

**Table 4:** Comparison of CEUS features of HCC with different pathological differentiation and tumor size according to contrast agent used.

Variable	Non-rim APHE		Rim APHE		Late (≥60 s) and Mild Washout		Early Washout (< 60 s)		No Washout		Kuppfer Phase Filling Defect
	Sonovue	Sonazoid	Sonovue	Sonazoid	Sonovue	Sonazoid	Sonovue	Sonazoid	Sonovue	Sonazoid	
Differentiation degree (n=52)											
Well-differentiated (n=2)	2 (100)	2 (100)	0 (0)	0 (0)	2 (100)	1 (50)	0 (0)	0 (0)	0 (0)	1 (50)	1 (50)
Moderately-differentiated (n=34)	32 (94.1)	32 (94.1)	2 (5.9)	2 (5.9)	25 (73.5)	23 (67.6)	9 (26.5)	10 (29.4)	0 (0)	1 (2.9)	34 (100)
Poorly- differentiated (n=16)	16 (100)	16 (100)	0 (0)	0 (0)	14 (87.5)	14 (87.5)	2 (12.5)	2 (12.5)	0 (0)	0 (0)	16 (100)
Tumor size (n=55)											
≤2 cm (n=11)	11 (100)	11 (100)	0 (0)	0 (0)	10 (90.9)	9 (81.8)	1 (9.1)	1 (9.1)	0 (0)	1 (9.1)	10 (90.9)
>2 cm (n=44)	42 (95.5)	42 (95.5)	2 (4.5)	2 (4.5)	34 (77.3)	32 (72.7)	10 (22.7)	11 (26.2)	0 (0)	1 (2.3)	44 (100)

Data are numbers of nodules with percentage in parentheses. CEUS, contrast enhanced ultrasound; HCC, hepatocellular carcinoma; APHE, arterial phase hyperenhancement.

Regarding the tumor size, there was no significant difference in the AP enhancement pattern between HCCs ≤2 cm and >2 cm on either SonoVue and Sonazoid imaging (with nonrim APHE seen in 53 of 55 (96.4%) HCCs ≤2 cm and rim APHE in two HCCs (3.6%) that were larger than 2 cm with both UCAs). Among the HCCs equal to or smaller than 2 cm, 10 of 11 (90.9%) and 9 of 11 (81.8%) HCCs exhibited late and mild washout on SonoVue and Sonazoid-enhanced US, respectively. One (1/11, 9.1%) HCC patient did not show washout during the vascular phase or KP defects on Sonazoid-enhanced US. This lesion was a WD HCC that was confirmed by histopathological analysis. In HCCs larger than 2 cm, 77.3% (34/44) of HCCs on SonoVue-enhanced US and 72.7% (32/44) on Sonazoid-enhanced US showed late and mild washout, respectively. One HCC (2.3%) measuring 4.5 cm did not show washout during the vascular phase, whereas it presented hypoenhancement in the KP. In addition, filling defects in KP were present in all HCCs larger than 2 cm.

### Diagnostic performance of CEUS for HCC

The sensitivity, specificity, and accuracy of the CEUS LI-RADS LR-5 category with the use of SonoVue and Sonazoid and the modified CEUS LI-RADS criteria (using KP defects as an alternative to late and mild washout in CEUS LI-RADS) with Sonazoid for HCC diagnosis are shown in **Table 5**. There was no significant difference between SonoVue-enhanced US and Sonazoid-enhanced US in diagnosing HCC, with the sensitivity being 80% [95% confidential interval (CI): 67%, 89.6%] and 74.6% (95% CI: 61%, 85.3%), respectively (*p* = 0.25), and the specificity being the same of 100% (95% CI: 59%, 100%) for both US agents (*p* = 1). The modified CEUS LI-RADS did not significantly increase the diagnostic efficacy of Sonazoid compared with that of CEUS LI-RADS, with a sensitivity of 76.4% (95% CI: 63%, 86.8%) and 74.6% (95% CI: 61%, 85.3%), respectively (*p* = 0.99). The modified CEUS LI-RADS LR-5 with the use of Sonazoid likewise achieved a specificity of 100% (95% CI: 59%, 100%).

**Table 5:** Diagnostic performances for hepatocellular carcinoma according to contrast agent used.

Variable	CEUS LR-5		*p* Value*	Modified LR-5	*p* Value†
	Sonovue	Sonazoid		Sonazoid	
Sensitivity	80 (44/55) [67, 89.6]	74.6 (41/55) [61, 85.3]	0.25	76.4 (42/55) [63, 86.8]	0.99
Specificity	100 (7/7) [59, 100]	100 (7/7) [59,100]	1	100 (7/7) [59, 100]	1
Accuracy	82.3 (51/62) [70.5, 90.8]	77.4 (48/62) [65, 87.1]	0.25	79 (49/62) [66.8, 88.3]	0.99

Data are numbers of nodules; data in parentheses are percentages. CEUS LI-RADS, contrast enhanced ultrasound Liver Imaging Reporting and Data System.

* The comparison between SonoVue-enhanced US and Sonazoid-enhanced US according to CEUS LI-RADS^®^ 2017.

† The comparison between Sonazoid-enhanced US by using CEUS LI-RADS^®^ 2017 and the modified criteria (using Kupffer-phase defects as an alternative to late and mild washout in CEUS LI-RADS).

### Safety

No adverse events were observed in any of the patients enrolled in this study.

## Discussion

This study individually compared SonoVue-enhanced US and Sonazoid-enhanced US for the diagnosis of HCC in patients at high risks and showed that they had comparable diagnostic efficacy for HCC. In the study conducted by Kang and his colleagues, the investigators found that Sonazoid-enhanced US had higher sensitivity than SonoVue-enhanced US in diagnosing HCC for patients at high risk (15). However, in another intraindividual study by the same team with expanded study population(n=105), the investigators found Sonazoid-enhanced US had noninferior efficacy to SonoVue-enhanced US for the diagnosis of HCC in at-risk patients. Besides, no significant improvement in HCC diagnosis was found when extending the washout time delay from 5 to 10 minutes on Sonazoid-enhanced US (25). In the current study, SonoVue-enhanced US had comparable diagnostic sensitivity and specificity with Sonazoid-enhanced US which is in concordance to the latest findings of Kang et al. Intriguingly, all HCCs had washout on SonoVue-enhanced US; however, two HCCs (3.6%, 2/55) did not have washout in the vascular phase on Sonazoid-enhanced US, and one of them did not even present a KP defect. The discrepancy may be due to the difference between the mechanism of washout of PBA and KPA. CEUS enhancement of HCC in the portal/late phase of PBA-enhanced US completely depends on the difference in portal vein blood supply between the liver parenchyma and lesions (26). However, late phase washout in KPA-enhanced US also depends on the difference in Kupffer cell uptake (17, 27). It is worth noting that phagocytosis of perflubutane microbubbles by Kupffer cells occurs even during the vascular phase, which inevitably contributes to the enhancement, although the importance of such a contribution is unknown (17).

Previous studies have reported that pathological differentiation of HCC is correlated with CEUS manifestation (27–30). WD HCCs are prone to later washout than moderately and PD HCCs, and the proportion of HCCs without washout is higher in WD HCCs than in more progressed HCCs. However, lesions without washout can also be found in moderate and PD HCCs (28, 30). In the current study, all HCCs had the same APHE with the use of both contrast agents. A WD HCC and an MD HCC did not show salient washout in the vascular phase on Sonazoid-enhanced US, and the WD HCC did not display defects in the KP; however, all HCCs had washout on SonoVue-enhanced US. This might be explained by the effect of Kupffer cell uptake, which obscured the observation of washout of the contrast agent. Moreover, Liu et al. reported that the number of Kupffer cells in WD HCCs is comparable to that in paracancerous tissue and adjacent normal liver tissue (31).

CEUS LI-RADS (version 2017) was designed for pure blood UCA only. The possibility of extending it to include KP UCA has been investigated. Hwang et al. found using KP defects as an alternative to late and mild washout in CEUS LI-RADS had a higher sensitivity than the established CEUS LI-RADS criteria for the diagnosis of HCC (18). However, pathological results were only obtained in 15.3% (31/203) patients which may comprise the strength of results. In our study, the modified criteria did not significantly improve the diagnostic performance of Sonazoid-enhanced US, whereas atypical hemangioma might be a pitfall for HCC. Atypical features include homogeneous APHE in small (15 mm) lesions or inhomogeneous APHE in large hemangiomas (> 4-7 cm) with arterio (porto-) venous shunts, sclerosing hemangiomas, and hemangiomas with regression changes (32). On KP imaging with Sonazoid, hemangiomas show iso- to hypoenhancement relative to the surrounding liver parenchyma and may mimic malignancies, including HCC (33, 34). Therefore, the washout time and degree in the portal phase on Sonazoid-enhanced US are also essential for the diagnosis of HCC, especially for patients at high risk.

Our study had several limitations. First, the sample size of the current study was relatively small. However, it is challenging to perform CEUS examination for a patient with the use of two different contrast agents. Second, the number of benign and non-HCC malignancies was comparatively insufficient. Further studies are needed to include the aforementioned entities to better understand the effect of UCA on the performance of CEUS on HCC diagnosis in patients at risk. Third, not all CEUS examinations were performed on the same US machine, which may lead to systematic bias to some extent. However, the US machine setup was restrictively followed according to the manufacturer’s instructions.

In conclusion, Sonazoid-enhanced US had comparable diagnostic performance with SonoVue-enhanced US for patients at high risk for HCC on the application of CEUS LI-RADS. KP of Sonazoid did not considerably improve the diagnostic efficacy, whereas the absence of KP defects in well-differentiated HCCs and KP hypoenhancement in atypical hemangioma may be pitfalls in diagnosing HCC. Further studies with larger sample sizes are needed to further validate the conclusions in the present study.

## Data availability statement

The original contributions presented in the study are included in the article/**Supplementary material**. Further inquiries can be directed to the corresponding author.

## Ethics statement

The studies involving human participants were reviewed and approved by Ethical Committee of West China Hospital. The ethics committee waived the requirement of written informed consent for participation. Written informed consent was obtained from the individual(s) for the publication of any potentially identifiable images or data included in this article.

## Author contributions

QL: Conceptualization, Methodology, Data review. JH: Writing- Original draft preparation, Methodology, Software. LG: Contrast enhanced ultrasound examination, Data collection and curation. JL: Data review. RY: Image processing. ZJ: Writing-Reviewing and Editing. ML: Literature retrieval. YL: Supervision. All authors contributed to the article and approved the submitted version.
