# Supplementary material for: Head-to-head comparison of Sonazoid and SonoVue in the diagnosis of hepatocellular carcinoma for patients at high risk
Source: Front Oncol. 2023 Mar 15;13:1140277. doi: 10.3389/fonc.2023.1140277 (PMC10050587; doi:10.3389/fonc.2023.1140277)
Supplement: Supplementary file 1 [file Table_1.docx]

**Supplementary material 1: CEUS examination protocol with the use of different UCA.**

- **SonoVue**: The contrast-enhanced ultrasound (CEUS) procedure was performed in accordance with the guidelines released by the European Federation of Societies for Ultrasound in Medicine and Biology after evaluation of nodules or observations by B-mode US. A low mechanical index (MI) of less than 0.1 was used for CEUS examinations with a dual screen format. Bolus injection of 1.2-2.4 mL of SonoVue (Bracco, Milan, Italy) was administered via the antecubital vein followed by a 5–10 mL normal saline flush at a rate of approximately 2 mL/s. The timer was simultaneously started as the ultrasound contrast agent (UCA) injection was completed. CEUS imaging was performed continuously from UCA injection until peak AP enhancement to capture peak AP enhancement and characterize the presence, intensity (hyper, iso, or hypo) and pattern of AP enhancement (diffuse, mosaic, rim, peripheral discontinuous globular). Otherwise, continuous imaging could be prolonged over peak AP enhancement until 60 s after UCA injection to determine early washout. After 60 s, imaging was performed intermittently (5–10 s every 30–60 s) to evaluate the presence and degree of late washout. Continuous images were recorded from the first bubble arrival through peak AP enhancement. Alternatively, the cine loop can be continued beyond the AP enhancement peak until 60 s after UCA injection. After 60 s, static images were recorded at 60 s, and intermittent (every 30–60 s) acquisition was performed to document and assess the presence, timing, and degree of washout.
- **Sonazoid**: For Sonazoid-enhanced imaging, a dose of 0.6-0.8 ml was used for UCA injection, and a low MI of 0.18-0.21 was set for CEUS examinations. Ten minutes approximately after CUA injection, imaging was recorded for several seconds. In addition to the aforementioned contents, the injection of UCA and operation in the vascular phases were identical to those of SonoVue CEUS imaging.

| **Supplementary Table 1: Pre-defined Settings of CEUS Examination According to UCA and US Machine Used** | | | | | | | |
| --- | --- | --- | --- | --- | --- | --- | --- |
| CEUS techniques | UCA | | | Dose of UCAs (mL) | MI | | |
|  | Microbubbles |  | Diameter (μm) |  | Philips EPIQ7 |  | Mindray Resona 7T |
| SonoVue-enhanced US | sulfur hexafluoride |  | 2.5 | 1.2-2.4 | 0.05 |  | 0.08 |
| Sonazoid-enhanced US | perfluorobutane |  | 3 | 0.6-0.8 | 0.18-0.21 |  | 0.18 |
| CEUS = contrast-enhanced ultrasound, UCA = ultrasound contrast agent, US = ultrasound, MI = mechanical index. | | | | | | | |

| **Supplementary Table 2: The Interobserver Agreements of CEUS Characteristics According to Contrast-enhanced Agent Used** | | | | | | | |
| --- | --- | --- | --- | --- | --- | --- | --- |
|  | APHE | |  | Washout Time and Degree | | Kupffer Phase Defect |  |
| Variable | Sonovue | Sonazoid |  | Sonovue | Sonazoid |  |  |
| Reviewer 1 vs. Reviewer 2 | 0.75 (0.45,1.0) | 0.71 (0.35, 1.0) |  | 0.82 (0.64, 0.99) | 0.78 (0.62, 0.94) | 0.91 (0.86, 1.0) |  |
| Note. —Data are κ values of interobserver agreements between reviewers, or between reviewers and operator, with 95% confidence intervals in parentheses. CEUS = contrast-enhanced ultrasound, APHE = arterial phase hyperenhancement. | | | | | | |  |
|  | | | | | | |  |
